# Chicago Medical Society

**Published:** 1869-10

**Authors:** 


### CHICAGO MEDICAL SOCIETY.

FRIDAY EVENING, *Sept. 3, 1869.*

The regular semi-monthly meeting of this Society was called to order, President R. G. Bogue in the Chair.

The Secretary read the minutes of the last meeting, which were duly approved.

In the regular order of business, the name of Dr. C. J. Lewis, was proposed for membership, by Drs. Ingalls and Wanzer.

Dr. Bogue, as Chairman of Committee to draft resolutions in case of the death of Dr. F. O. Earle, presented and read said resolutions, which were accepted by the Society with the provision, that the resolutions be published in the city papers, and a copy be furnished the family of the deceased.

The discussion of the "Pathology and Treatment of Hæmoptysis," was opened by Dr. Hurd, who gave a detailed and interesting account of the symptoms connected with hemorrhage from the lungs. Thinks if there was clotted blood in the lungs there would be dulness on percussion, but *post mortems* generally show that the blood is infiltrated into the lobules. Is of the opinion, that in the majority of cases there is no special lesion of the coats of the artery, but where from tuberculous deposit it comes from the vessel itself and not from transudation. Considers hemorrhage merely a symptom that generally occurs before the softening stage, the tubercle acting as a foreign and irritating body. Thinks there is less heart disease in cases of hæmoptysis than Trousseau would have us believe. Carcinoma of the lungs may also be a cause as well as thoracic aneurism, pneumonia, and gangrene of the lungs.

Thinks the only thing we are apt to confound it with is *hœmatemesis*, although in this condition the blood vomited is blackened and decomposed. If the blood only tinges the sputa, it may come from the pharynx. Also spoke of the occasional occurrence of vicarious menstruation which might be the cause of the hemorrhage.

He considers the prognosis very bad, as it is generally due to tubercle. Treatment, gives plumbi acets and opii frequently; and Aitken recommends potassa bitart. ʒj.; P. opii gr. j., given often and continued for forty-eight hours. If there is labored action of the heart, arterial sedatives. The French use leeches to chest. He also spoke of Warren's styptic —sulphuric acid dropped into alcohol, and a sol. ferri per sulphates, but he did not like the action of the last-named remedy.

If the hæmoptysis be due to vicarious menstruation, then determine the blood to the uterus; recommends argenti nitras to be applied to the os uteri.

Dr. Marguerat thinks there is no disease more trying to the physician. As regards the prognosis. Says there is great diversity of opinion, some contending that nine-tenths, while others not more than one-half of the cases of hæmoptysis are due to tuberculosis. Says he saw one case where there was no signs of tubercle. Of the treatment, Dr. M. considers the French too heroic, as they bleed too much. Says if we adopt the theory that many cases are not due to phthisis, but congestion, we should bleed. Says Dr. Watson leeches in these cases. has not much confidence in medicine to relieve the hemorrhage, but depends more on quiet, cooling drinks, and good fresh air. Says he has never had a case of fatal hæmoptysis, and that Dr. Flint says he has never seen a case of vicarious menstruation in his whole practice, hence is of the opinion that it is of rare occurrence.

The Doctor referred to the case of a young man whom he was called to see, a few weeks since. It was at night, and when he arrived he found that the patient had spit up two or three teaspoonsful of blood. Could detect no signs of phthisis at the time, but nevertheless the young man died of phthisis in two weeks.

Has at present a young lady under his care, who suffers from hæmoptysis. The lady is fleshy and rosy, and does not look at all like a tuberculous subject, but nevertheless ascultations reveals metallic tinkling over a small spot posteriorly.

Dr. Frediegke thinks we are apt to find many of these patients plethoric, and, in such cases, thinks that veratrum viride, in full doses, accompanied by astringents, may be found very beneficial.

Dr. Paoli remarked, that we have to be very careful in our decision, for tubercle may exist in the lung, while the tissue external to the deposit may be healthy; hence, percussion is clear, and ascultation good, *post mortem* only revealing the presence of tubercle.

The Dr. further said, that the distinction between hæmoptysis and hæmatemesis was, that in the former, the pulse was hurried and excited, while in the latter it is slower, and the face is congested. Cited a case which he saw in Small-Pox Hospital, some 12 years ago. Patient suffering from cirrhosis of the

40

liver, when there was hæmoptysis to the amount of 12 ounces. Lungs dull on percussion. On *post mortem*, there was found to be, in addition to the cirrhosis of the liver, calculi of the ducts; while the hemorrhage seemed to have been entirely from the larynx.

Of the treatment: does not recommend bleeding, in the asthenic cases. Thinks gallic acid, in 10 or 12-gr. doses, every half hour, far preferable to the opii and plumbi acetatis, as it does not tend to constipate the bowels. The pulse may be diminished by digitalis, keeping patient quiet, and administering cool drinks. Says he has never seen a case die, but admits there are no cases more perplexing and alarming to the physician than those of hæmoptysis.

Dr. Fitch said, his experience had not been very great, but the majority of cases of hæmoptysis that had come under his observation had resulted in tuberculosis. Had never treated a case of hæmoptysis in a plethoric subject, but if he were called in such a case, he should not hesitate to bleed. The usual treatment employed by him consists in elevating the shoulders; ice to chest; gallic, or tannic acid, or lead and opium internally; and, in some cases, has seen great benefit derived from ergot, with sinapisms to the extremities and acidulated cold drinks.

Dr. Merriman remarked, that his experience had, also, been quite limited, but that he had seen several cases of great interest. Was called one night last winter to see a man who was bleeding from the lungs; but, when he arrived, found the patient dead. Patient had complained of a feeling of oppression in the chest, followed by a copious gush of blood over the side of the bed. This was a tuberculous patient.

Another was in the case of a rosy-cheeked, plethoric young lady, who was dressing to go to a party, when she was suddenly attacked with bleeding from the lungs. Dr. says he was in the next house, and was summoned immediately. When he arrived, found the woman's garments and bed saturated with blood, the woman having completely fainted away. Judges the amount of blood lost to be from 12 to 16 ounces.

In a week, she had another attack, and lost 6 or 8 ounces of blood, and continued to have them for six or eight weeks, losing from 2 to 8 ounces each time. The hemorrhage occurred about three years ago, and the lady is now married, the mother of a fine healthy baby, and in perfect health herself. The treatment in this case consisted in the administration of plumbi acetatis, and opii, and sulphuric acid and iron, latterly, with good hygienic treatment.

The discussion was also participated in by Drs. Hutchinson, Bridge, and others. The President announcing the discussion closed.

The committee ordered to draft resolutions, in case of the death of Dr. D. D. Waite, consisting of Drs. Davis, Loverin, and Paoli, rendered the same, which was read before the Society, and accepted, with the provision that the resolutions be published in the city papers, and a copy of the same be furnished the family of the deceased.

Dr. Davis remarked, that he had known Dr. Waite a good many years, and spoke in the highest terms concerning his ability and character. Said that while Dr. Waite was President of the Chicago Medical Society, it was never more prosperous; and that it was due to the energy of their President, who left it in a more flourishing condition than it had been for yeaas. Dr. D. further remarked, that owing to Dr. Waite's ill health, we have not seen much of him of late years, but the older members of the Society will remember him as one of their most attentive members, and earnest, hard workers.

Dr. Paoli also made equally complimentary remarks.

Dr. Marguerat said, that the last time he saw Dr. Waite was about one year and a-half since, when he saie to Dr. M.: "Tell my friends in the Society that I am with them, if not in person, in heart and soul."

Dr. Loverin also made complimentary remarks.

The Society then proceeded to miscellaneous business.

